# Pathogenesis and Clinical Management of Mesenteric Fibrosis in Small Intestinal Neuroendocine Neoplasms: A Systematic Review

**DOI:** 10.3390/jcm9061777

**Published:** 2020-06-08

**Authors:** Anna Koumarianou, Krystallenia I. Alexandraki, Göran Wallin, Gregory Kaltsas, Kosmas Daskalakis

**Affiliations:** 1 Hematology Oncology Unit, Fourth Department of Internal Medicine, Attikon University General Hospital, National and Kapodistrian University of Athens, 12462 Athens, Greece; 2 1st Department of Propaedeutic Internal Medicine, Endocrine Unit, National and Kapodistrian, University of Athens, 11527 Athens, Greece; alexandrakik@gmail.com (K.I.A.); gkaltsas@endo.gr (G.K.); kosmas.daskalakis@oru.se (K.D.); 3 Department of Surgery, Faculty of Medicine and Health, Örebro University, 701 85 Örebro, Sweden; goran.wallin@regionorebrolan.se

**Keywords:** neuroendocrine tumors, small intestine, mesenteric fibrosis, serotonin, TGF, FGF, PDGF, VEGF, CTGF

## Abstract

Mesenteric fibrosis (MF) constitutes an underrecognized sequela in patients with small intestinal neuroendocrine neoplasms (SI-NENs), often complicating the disease clinical course. The aim of the present systematic review, carried out by Preferred Reporting Items for Systematic Reviews and Meta-Analyses (PRISMA) methodology, is to provide an update in evolving aspects of MF pathogenesis and its clinical management in SI-NENs. Complex and dynamic interactions are present in the microenvironment of tumor deposits in the mesentery. Serotonin, as well as the signaling pathways of certain growth factors play a pivotal, yet not fully elucidated role in the pathogenesis of MF. Clinically, MF often results in significant morbidity by causing either acute complications, such as intestinal obstruction and/or acute ischemia or more chronic conditions involving abdominal pain, venous stasis, malabsorption and malnutrition. Surgical resection in patients with locoregional disease only or symptomatic distant stage disease, as well as palliative minimally invasive interventions in advanced inoperable cases seem clinically meaningful, whereas currently available systemic and/or targeted treatments do not unequivocally affect the development of MF in SI-NENs. Increased awareness and improved understanding of the molecular pathogenesis of MF in SI-NENs may provide better diagnostic and predictive tools for its timely recognition and intervention and also facilitates the development of agents targeting MF.

## 1. Introduction

Small intestinal neuroendocrine neoplasms (SI-NENs) comprise a rare tumor entity with rising incidence, but overall favorable survival outcomes despite frequent presentation at distant stage disease [1]. They originate in enterochromafin (EC) cells, usually in the terminal ileum and less commonly in the jejunum. The primary tumor is typically submucosal and < 1cm in size, whereas in up to 50% of patients multifocal tumors can be found arising independently in the small intestine [2,3]. Mesenteric lymph node (LN) metastases are commonly present at diagnosis and tend to grow conspicuously large and often induce mesenteric fibrosis (MF) in the surrounding tissue of the mesentery.

The release of various amines, peptides and growth factors (GFs) may induce hormonal symptoms that constitute the carcinoid syndrome (CS), but also fibrosis in cellular systems leading to MF and carcinoid heart disease (CHD) [4,5]. In addition, neuroendocrine neoplasm (NEN) cells overexpress a plethora of proangiogenic molecules that promote both recruitment and proliferation of endothelial cell precursors becoming highly vascularized neoplasms [6].

Locoregional LN metastases together with the accompanying MF may occur in up to 50% of SI-NENs with encasement of the superior mesenteric vessels at different levels in the mesentery [7,8]. Locoregional resective surgery when feasible constitutes the mainstay of treatment in patients with MF-related symptoms. Although advances in systemic and liver-targeted treatment of SI-NENs have been made, alleviating hormonal symptoms and prolonging progression-free (PFS) and overall survival (OS), effective treatment options targeting MF are not fully elucidated. As improved insight in the complex pathogenesis of MF is key to the development of new therapies, we have reviewed evolving aspects of tumor microenvironment (TME), mediators and signaling pathways involved in the molecular pathogenesis of MF in SI-NENs, in order to refine the management of patients suffering from fibrotic complications.

## 2. Experimental Section

**Figure 1:**
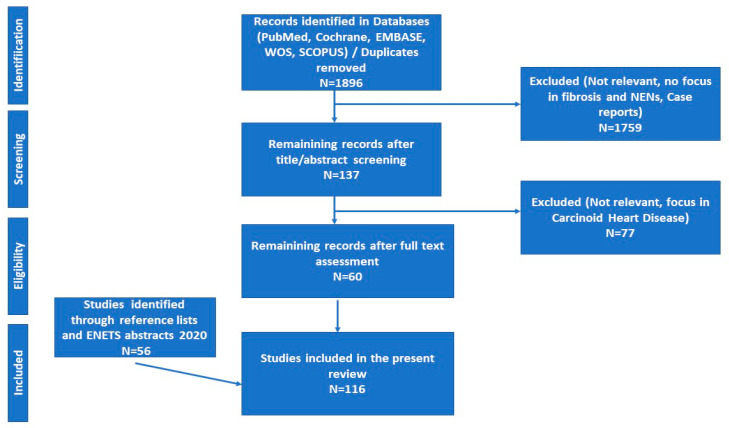
Preferred Reporting Items for Systematic Reviews and Meta-Analyses (PRISMA) flow diagram of study selection. Abbreviations: ENETS; European Neuroendocrine Tumor Society; NENs: Neuroendocrine Neoplasms.

We performed a systematic review of the literature available on PubMed, Cochrane Library, Embase, Web of Science and SCOPUS databases about MF in SI-NENs until March 15th, 2020, as specified in the “Search Strategy Supplement” (Table S1). Articles were independently evaluated by two of the authors (K.D. and K.I.A.) for the relevance to the planned scope of the review. Reference lists of key publications were also reviewed for eligibility. We followed Preferred Reporting Items for Systematic Reviews and Meta-Analyses (PRISMA) guidelines for reporting [9]. The literature search and the selection of included studies are presented in the PRISMA flow diagram (Figure 1).

## 3. Results

### 3.1. Pathogenesis of Mesenteric Fibrosis (MF)

In SI-NENs, MF represents an abnormal repair process, mimicking the mechanism operating in certain types of connective tissue disorder, associated with serious complications during the course of the disease [10,11]. Serotonin and vasoactive substances were the first effectors to be implicated in the pathogenesis of CS and into its more severe complications, the MF or CHD, representing the fibrotic-related sequelae of CS. There is currently evidence that CHD pathophysiology is mostly based on circulating effectors, specifically on high serotonin levels as depicted by the high urine level of 5-hydroxyindoleacetic acid (5-HIAA), whereas MF seems to be caused by a complex network of autocrine and paracrine mediators produced by neoplastic cells and TME elements [11].

TME represents an important functional unit comprising a cluster of endothelial and inflammatory cells and mesenchymal stroma elements with fibroblasts. This network of cells is interconnected and interacting, leading to tumor growth and development of fibrosis, possibly through epigenetic changes of tumor cells [6,12,13]. TME has been recently depicted as a dynamic milieu where an injury with fragmentation of extracellular matrix elements results in chemo-attractant molecules recruiting immune cells that promote a tumorigenic environment, followed by pro-angiogenic elements during tissue repair.

Proteomic analysis of SI-NENs and associated MF primarily revealed differences in mesenteric stroma showing that certain proteins are involved in the formation and regulation of the extracellular matrix and the complement cascade [14]. In addition, a multitude of immune cells including B and T cells, natural killer (NK) cells, mast cells, dendritic cells and macrophages have been reported to infiltrate NENs, resulting in an immunosuppressed TME, which promotes tumor growth [6]. NEN patients display immune recognition of their tumors, as CD8+ T-cells target specific SI-NEN-associated antigens including chromogranin A and tryptophan hydroxylase [15]. Recently, several studies have addressed the expression of programmed death-ligand 1 (PD-L1) as a potential mechanism of NEN immune evasion, with the upregulation of PD-L1 exhibited in a subset of SI-NENs [16,17,18].

Importantly, in vitro studies in currently available pancreatic NEN cell lines cannot serve as an ideal model for assessment of MF. However, the development of three-dimensional organoids/spheroids may be a promising model to study the SI-NEN stroma complexity and its influence in epithelial tumorigenesis in the context of MF. Recently, SI-NEN cells have been cultured from surgically removed tumors as spheroids in ECM, forming a three-dimensional matrix that encapsulates SI-NEN cells and mimics TEM [19]. Although, organoid/spheroid systems lack some important components present in vivo, such as fibroblasts, endothelial cells and immune cells, coculture of organoids with other cell types has been attempted in order to generate a more “physiological” TEM, and to study cell–cell interactions with potential future implications in the study of MF [20].

**Figure 2:**
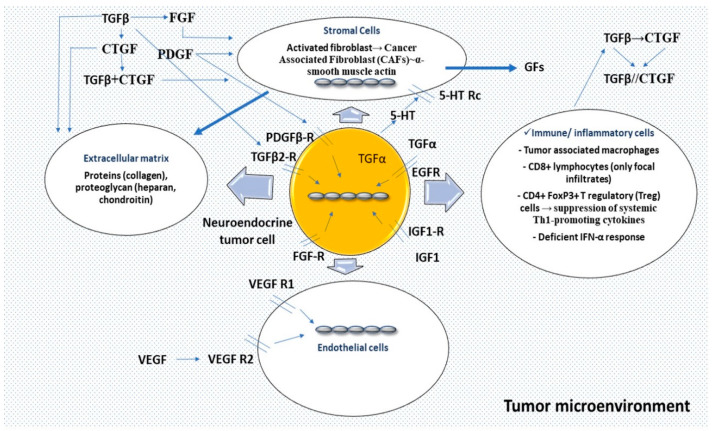
Tumor microenvironment represents an important functional unit comprising a cluster of endothelial and inflammatory cells, and mesenchymal stroma elements with fibroblasts interconnected and interacting. An initial event, an injury, induces fragmentation of extracellular matrix elements, chemo-attractant molecules with recruitment of immune cells acting as a pro-tumorigenic environment, followed by pro-angiogenic elements. In specific circumstances, serotonin (5-HT) via a receptor (5-HT Rc) may be mitogenic in stroma cells acting through a TGFβ-mediated mechanism or potentiating the effects of PDGF, β-FGF, or EGF and insulin. TGF-β represents a pro-fibrotic mediator; TGFβ1 and the receptor subtype-2 (TGFβr2) have been identified in SI-NEN. CTGF is transcriptionally activated principally through TGFβ1, acting as a downstream mediator of its profibrotic activities on fibroblasts; PDGF, EGF and FGF are also activating CTGF gene expression at the transcriptional level. PDGF is a mitogen for the fibroblasts and the smooth muscle cells. Infiltration of immune cells is not high in SI-NENS, but tumor-associated macrophages suppress the adaptive immune system and stimulate fibrosis by secretion of profibrotic factors such as TGFβ. Abbreviations: 5-HT: 5-hydroxytryptamine; 5-HT Rc: 5-hydroxytryptamine receptor; TGFβ: transforming growth factor-β; PDGF: platelet-derived growth factor; β-FGF: β-fibroblast growth factor; EGF: Epidermal growth factor; TGFβr2: transforming growth factor-β receptor subtype-2; CTGF: connective tissue growth factor; CAF: cancer-associated fibroblast; GFs: growth factors; IGF-1: insulin growth factor type 1; VEGF: vascular endothelial growth factor; VEGF R: vascular endothelial growth factor receptor; CD8: cluster of differentiation 8; CD4: cluster of differentiation 4; FoxP3: forkhead box P3; Th1: T helper type 1; IFN-α: Interferon alpha.

The effort to understand TME elements sheds light on the paracrine role that GFs may exert, affecting the molecular pathways implicated in fibroblast growth, proliferation and action [12,21]. A number of mediators have been shown to play an essential role in MF pathogenesis (Table 1 and Figure 2).

#### 3.1.1. Serotonin

Serotonin (5-hydroxytryptamine; 5-HT) has been classically considered as the main mediator of MF in SI-NENs. However, no consistent association has been documented between MF and elevated serotonin levels [10,56]. Serotonin exhibits both mitogenic and fibrogenic effects in fibroblasts, smooth muscle cells and endothelial cells.

The strongest evidence that implicates serotonin in the interplay of fibrosis is the finding of higher levels of 5-HIAA in patients with CHD compared to patients without cardiac involvement [57,58,59]. Serotonin administration induces CHD through a transforming growth factor beta (TGFβ)-mediated mechanism [60], and the presence of serotonin likely modulates connective tissue growth factor (CTGF) function. Moreover, serotonin acts in association with other molecules, potentiating the mitogenic activity of the β-fibroblast growth factor (FGF) and the combined action of epidermal growth factor (EGF) and insulin [26].

Transcript levels and the secretion of TGβ1, CTGF and FGF2 were significantly reduced in SI-NEN treated with a specific 5-HT2B receptor antagonist in vitro [12]. Thus, 5-HT2B receptors and serotonin have an important role in the regulation of peri-tumoral fibrosis and angiogenesis, sustaining TME [12]. In addition, the MEK/extracellular signal-regulated kinase (ERK) pathway is potentially playing a role in this process, since serotonin acts via the G-protein coupled 5-HT receptors, which are activating mitogenic pathways [12].

Another mechanism for the pathogenesis of MF may be the decreased expression of serotonin-degrading enzymes in the stromal compartment of mesenteric metastases [61]. Recently, the tryptophan metabolism pathway was investigated by label-free proteomics in MF. Quantification of h-5-HT and h-5-HIAA in blood and tissues was simple, highly specific and predictive of steady state changes in 5-HT, opening the possibility for rapid screening of TPH1 inhibitor dose response, such as that of telotristat etiprate [62].

#### 3.1.2. Growth Factors (GFs)

GFs act locally by a combination of paracrine and autocrine actions to stimulate cell proliferation and differentiation. Platelet-derived growth factor (PDGF), insulin growth factor 1 (IGF-1) and -2, EGF, TGF-α and TGF-β have shown mitogenic properties on fibroblasts [47].

##### Transforming Growth Factor-β (TGF-β)

TGFβ can stimulate both the production and deposition of the extracellular matrix via increased expression of collagen, fibronectin, proteoglycan and integrin expression in parallel to decreased expression of proteases and increased expression of protease inhibitors [63]. TGFβ is also chemotactic for fibroblasts and macrophages and may induce a rapid local fibrotic response [28]. TGFβ1 and the receptor subtype-2(TGFβrII) have been identified in SI-NEN samples, confirming the interaction of tumor cell with TME to exert its pro-fibrotic role in NENs [31].

##### Connective Tissue Growth Factor (CTGF)

CTGF is involved in the coordination of complex biologic processes such as differentiation, tissue repair and angiogenesis. The low-density lipoprotein (LDL) receptor-related protein/α-2-macroglobulin receptor (LRP2) has been demonstrated to be the receptor for CTGF in fibroblasts [64]. By signaling via integrins, a mechanistic interpretation is provided for the chemotactic and mitogenic properties of CTGF, as well as for its functions in ECM remodeling during angiogenesis and tissue repair [65]. CTGF is transcriptionally activated principally through TGFβ1 [40]. However, PDGF, EGF and FGF also activate CTGF gene expression at the transcriptional level [41,66]. Processes such as TGFβ-induced fibroblast proliferation, collagen synthesis and myofibroblast differentiation are regulated by CTGF-dependent pathways. Consequently, CTGF represents a downstream mediator of the profibrotic activities of TGFβ1 acting on fibroblasts, being a mediator of MF in SI-NENs [10,37,38].

SI-NEN tissues express high CTGF levels [67]. Proteomic and tissue microarray analysis demonstrated that SI-NENs synthesized and secreted CTGF. Serum levels of both CTGF and TGFβ1 were significantly increased in SI-NENs, suggesting that CTGF could be a marker for MF in SI-NENs [67,68]. Overall, TGF-β1 has an autocrine role in SI-NENs besides its paracrine role in MF by inducing CTGF [31]. The CTGF expression pattern is also correlated with the severity of MF.

All these data suggest that an MF network operates, where the initial event is secretion of TGFβ1, which in turn induces CTGF to act in conjunction, thus leading to the overproduction of collagen after bypassing tissue repair suppression. This network seems to be even more complex since other molecules are possibly participating (Figure 2). In vitro experiments have also demonstrated that the N-terminal region of CTGF stimulates myofibroblast differentiation and collagen production, while the C-terminal stimulates fibroblast cell proliferation [37]. CTGF can be found in SI-NEN patient serum and it is potentially involved in MF [69].

##### Platelet-Derived Growth Factor (PDGF)

SI-NENs express PDGF chains and PDGF receptor types [42]. An autocrine growth may be justified by α-receptors, whereas, tumor cells express B- and A-chains, which may also be synthesized by stromal cells stimulating tumor growth in a paracrine manner [42,43]. PDGF was found to be expressed in tumor cells and stroma in SI-NENs. PDGF α-receptors were found on tumor cells and adjacent stroma, whereas PDGF β -receptors were found only in stroma. Hence, PDGF may be involved in the autocrine stimulation of tumor cells and stimulation of stromal cell growth and matrix deposition.

Cryosections from SI-NENs showed that the PDGF β-receptor is expressed in 66%, as opposed to 10% of non-NENs tissues respectively. The immunoreaction was present in connective tissue cells adjacent to tumor cell clusters, but not in the tumor cells per se and were more intense in the proximity to the tumor compared to more distant foci [13]. This finding implies that SI-NEN cells may directly or indirectly induce expression of the PDGF-β receptor on adjacent stromal cells in the tumor tissue, which may possibly contribute to the stimulation of connective tissue cell proliferation in SI-NENs [10,13]. However, in one study no detection of PDGFR expression in SI-NENs was reported [46]. In parallel with this, a phase-II study of imatinib, specifically inhibiting the tyrosine kinase domain in PDGFR, showed no substantial regression of SI-NEN, but a significant number of patients with progressive disease achieved disease stabilization [44].

##### Insulin-Like Growth Factor 1 (IGF-1)

Insulin growth factor 1 plays an important role in the physiological regulation of cell growth and differentiation. IGF-1 receptor is present in SI-NEN, suggesting that it may possibly act as an autocrine stimulator of tumor growth [36,37,46]. The recent marker IGF-IEc, an isoform produced by the transcripts of the alternative splicing of the IGF-1 gene has been found to be expressed in SI-NENs [45]. However, since it is in an in vivo SI-NEN model, the serotonin-IGF-1 axis was found to act differentially depending on serotonin levels; hence, more focused studies on MF have yet to be performed [48].

##### Epidermal Growth Factor (EGF) and Transforming Growth Factor-a (TGF-α)

EGF in addition to the transforming growth factor alpha (TGF-α) and -β families of GFs, can stimulate or inhibit proliferation and differentiation in multiple tissues [70]. TGF-α was found to be abundant in tumor cells mediating its effects by binding to the EGF receptor (EGF-R) [47]. TGF-α and EGF receptors are expressed in SI-NENs [46,47], supporting the role of TGF-α in the autocrine regulation of tumor growth [49]. The presence of EGF-R in the stromal component of SI-NENs potentially implies the enhanced role of EGF in the process of MF.

##### Other Growth Factors (GFs)

FGF 2 is a well-known stimulator of vascular endothelial cells and β-FGF is a potent endothelial cell mitogen, playing a crucial role in tumor angiogenesis [12].

Her2/neu and vascular endothelial growth factor (VEGF)-receptors (VEGF-Rs) were overexpressed in SI-NENs [46]. More recently, it was demonstrated that interleukin-6 (IL-6), VEGF and monocyte chemo-attractant protein 1 (MCP-1) are actively secreted by cancer-associated fibroblasts, to induce tumor cell proliferation [6]. VEGF expression in vitro was modulated by oxygen levels through a pathway regulated by hypoxia-inducible factor 1 (HIF-1α) [50]. In addition, human fibroblasts have been shown to secrete nerve growth factors (NGFs) that have regulatory effects on angiogenesis [36]. In another study, the fibrotic marker vascular adhesion protein-1 (VAP-1) was highly expressed in SI-NEN stroma in close association with the extracellular matrix [51].

In summary, TME represents an important functional unit comprising a cluster of endothelial and inflammatory cells and mesenchymal stroma elements with fibroblasts interconnected and interacting. Fragmented extracellular matrix elements and chemo-attractant molecules recruit immune cells that act as a pro-tumorigenic environment, interacting with pro-angiogenic elements. In specific circumstances, 5-HT via its receptor may be mitogenic in stroma cells acting through a TGFβ-mediated mechanism or potentiating the effects of PDGF, β-FGF or EGF and insulin. CTGF is transcriptionally activated principally through TGFβ1, acting as a downstream mediator of its profibrotic activities on fibroblasts, whereas PDGF, EGF and FGF are also activating CTGF gene expression at the transcriptional level. Of note, infiltration of immune cells is not high in SI-NENS, but tumor-associated macrophages may display a tumor-promoting role whilst they suppress the adaptive immune system and stimulate fibrosis by secretion of profibrotic factors such as TGFβ (Figure 2).

### 3.2. Clinical Presentation

#### Symptoms and Related Deficiencies

Patients with SI-NENs are rarely diagnosed before locoregional LN metastases have developed, and often present with advanced symptomatic disease. In this setting, the disease clinical manifestations are most commonly due to MF around locoregional tumor deposits. MF can cause contraction and tethering of the adjacent bowel loop leading to acute symptoms, such as intestinal obstruction, intussusception and/or intestinal ischemia by impaired blood supply to the intestines [71,72]. The presence of advanced MF associated with intestinal ischemia has been recently identified as a poor prognostic factor for OS [5]. Venous ischemia due to superior mesenteric vein involvement is more often encountered as compared to its arterial counterpart, resulting in venous stasis, abdominal pain, aggravated diarrhea, ascites, malabsorption and malnutrition [73]. In advanced cases, MF surrounding LN metastases in the root of the mesentery may lead to shrinkage and fixation of it to the retroperitoneum that can cause in turn obstruction of the small intestine, duodenum and/or transverse colon. Chyloabdomen may also occur in patients with advanced MF owing to lymphatic obstruction. Occasionally, CS may accompany MF with retroperitoneal extension, when tumor secretory products exceed the detoxifying capacity of the liver, or bypass it, draining directly into the systemic circulation through retroperitoneal lymphatic spread [4,5,71,74]. Extensive retroperitoneal fibrosis may be complicated with obstructive uropathy, hydronephrosis and renal failure late in the disease clinical course [5]. Moreover, involvement of the vasculature in the root of the mesentery may result in collaterals that could predispose for lower gastrointestinal hemorrhages.

Apart from acute symptoms, MF can cause partial bowel obstruction and chronic postprandial abdominal pain, influencing patients’ food intake and nutritional status in the long term. In addition, profound diarrhea due to refractory CS, but also due to venous stasis and malabsorption in cases with extensive MF may induce nutritional deficiencies and quality of life impairment. As part of malnutrition in this setting, there are often encountered deficiencies in fat-soluble vitamins, mainly vitamin D, whereas severe disturbances in water and electrolyte homeostasis may also occur [75]. Moreover, in patients with CS, up to 60% of tryptophan is used for serotonin production leading to tryptophan deficiency and subsequently lower niacin levels and sometimes pellagra in a subset of patients [75,76,77]. However, although malnutrition in cancer patients can result in low levels of trace elements like cobalt, copper, fluorine, iodine, selenium and zinc, little is known about trace elements’ deficiencies owing to aggravated diarrhea related to MF in patients with SI-NENs [75].

### 3.3. Diagnostic and Classification Systems

**Figure 3:**
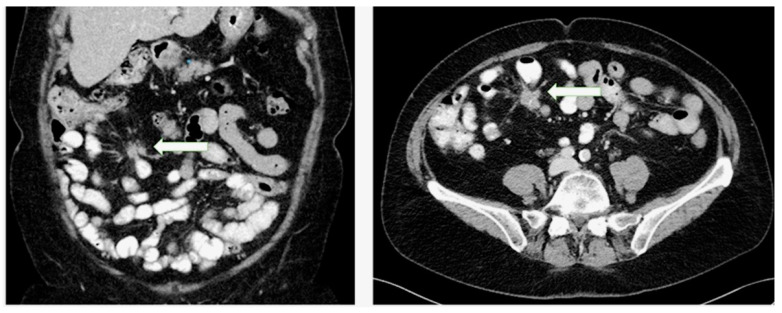
Presence of mesenteric fibrosis in a patient with a small intestinal neuroendocrine tumor as an enhancing soft-tissue mass with fibrotic bands radiating outward in the mesenteric fat in a stellate pattern around a lymph node metastasis in coronary and transverse planes.

The presence of MF is radiologically defined as enhancing spiculated soft-tissue mass with fibrotic bands radiating outward in the mesenteric fat in a stellate pattern around an LN metastasis on cross-sectional imaging [72,78] (Figure 3). The radiological severity of MF is based on the following classification: (a) no radiological evidence of MF (absence of radiating strands), (b) mild MF (≤ 10 thin radiating strands), (c) moderate MF (> 10 thin strands or < 10 thick strands) and (d) severe MF (≥ 10 thick strands) [78]. Of note, the radiological severity described by Pantograg—Brown is not correlated with any kind of clinical aspects such as symptoms or prognosis [78]. The histological assessment of MF is based on the histological section of the surgical specimen with the maximum amount of fibrous tissue. The mesenteric LN metastasis and surrounding tissue are examined for MF using Sirius Red staining and the width of the thickest fibrous band surrounding the tumor is measured.

Importantly, the extension of mesenteric LN metastases and associated MF below or above the horizontal part of the duodenum is a crucial factor for surgical planning [79]. An arbitrary staging system of the mesenteric LN metastases and accompanying MF has been developed by the Uppsala group (Stage I: LN metastases close to the intestine; Stage II: LN metastases higher in the mesentery; Stage III: LN metastases along, but not encasing the mesenteric vessels at the level of the horizontal duodenum; Stage IV: LN metastases extending above the horizontal part of the duodenum, encasing the superior mesenteric vessels) [8]. However, the validity of this system and its effect on surgical management of SI-NEN patients has not been established to date.

Notably, when utilizing histopathological, surgical and radiological correlations, computed tomography (CT) imaging has limitations and may underestimate the presence of MF [80,81]. This needs to be considered when making clinical decisions regarding the management of these patients in view of the considerable morbidity associated with MF. Importantly, as most SI-NENs express somatostatin receptors on the cell surfaces, functional imaging with somatostatin receptor-based Gallium-68 positron emission tomography–computed tomography (^68^Ga–PET–CT) has been shown to detect more primary SI-NENs and also metastatic tumor deposits, including peritoneal carcinomatosis lesions frequently encountered in the proximity of advanced MF, as compared to conventional contrast-enhanced CT [5,82]. Therefore, functional imaging with ^68^Ga-PET–CT may have a role in the accurate detection and staging of lymph node metastases surrounded by MF with important implications in surgical planning. On the other hand, fluorine-18 ((18)F)-fluorodeoxyglucose ((18)F-FDG) PET may be of limited value in low-proliferative well-differentiated SI-NENs. However, it could be particularly helpful for visualizing a subset of more aggressive SI-NENs, such as well-differentiated tumors with Ki67 values > 10%. According to limited data, (18)F-FDG-avid tumor lesions, even in slow-growing NENs, may indicate a more aggressive biological behavior [83]. Nevertheless, as available pathology data from SI-NEN patients with MF fibrosis have confirmed fibrotic reactions in close proximity to mesenteric LN metastases [5], molecular imaging studies along with conventional cross-sectional imaging can be used to delineate the management of complex cases with MF.

When considering the imaging modalities used in other fibrotic conditions, such as liver fibrosis, current cross-sectional and functional methods of evaluating its extent often provide an incomplete picture, whereas certain methods such as ultrasonography and elastography are not applicable in the setting of MF. A novel PET tracer has recently been proposed in a preclinical study demonstrating that imaging hepatic integrin αvβ3 with PET and (^18^F)-Alfatide may offer a non-invasive method for monitoring the progression of liver fibrosis [84].

With respect to diagnostic biomarkers for MF, only a few studies have assessed the utility of non-invasive monoanalyte biomarkers (serum CTGF and urinary 5-HIAA) in this setting [69,85,86]. However, these markers perform modestly and have not been specifically applied in the clinical practice for the assessment of MF as yet. Recently, a set of five profibrotic circulating transcripts with known roles in fibrosis (CTGF, CD59, amyloid precursor-like protein 2 (APLP2), frizzled homologue (FZD) 7 and BCL2/adenovirus E1B 19 kDa protein-interacting protein 3-like (BNIP3L)), taking the name of “fibrosome” has been assessed [81]. The combination of these five circulating transcripts (from the entire 51-gene molecular signature of the NETest) has demonstrated an accuracy of 100% for the detection of microscopic MF and a higher accuracy, as compared to radiological and surgical assessments. Given the ability of the multianalyte NETest to act as a liquid biopsy that can capture the multidimensionality of NEN, the “fibrosome” set appears to be a promising biomarker for MF detection [81]. However, further validation of these results is warranted in prospective studies.

### 3.4. Clinical Management of Mesenteric Fibrosis

#### 3.4.1. Surgical Management

**Figure 4:**
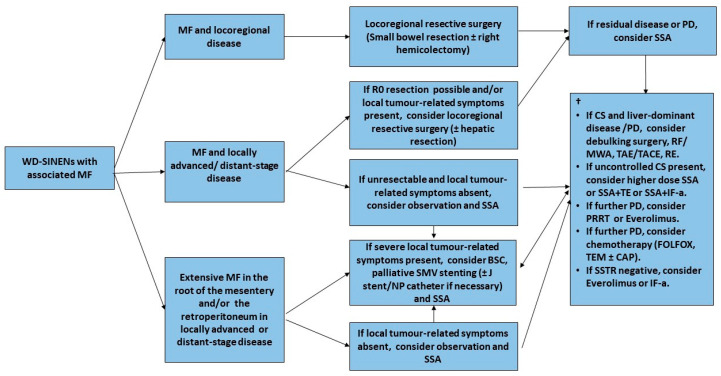
Management of Small Intestinal NENs with Mesenteric Fibrosis-Flow diagram. † Systemic treatment recommendations adapted from ESMO 2020 Clinical Practice Guidelines for diagnosis, treatment and follow-up of Gastroenteropancreatic Neuroendocrine Neoplasms [96]. Abbreviations. BSC: best supportive care; CAP: capecitabin; CS: carcinoid syndrome; FOLFOX: Folinic acid, Fluorouracil (5-FU) and Oxaliplatin; IF-a: interferon alpha; MF: mesenteric fibrosis; MWA: microwave ablation; NP: nephropyelostomy; PD: progressive disease; PRRT: Peptide Receptor Radionuclide Therapy; RE: radio-embolization; RF: Radiofrequency ablation; TACE: trans-arterial chemo-embolization; SMV: superior mesenteric vein; SSA: somatostatin analogue; SSTR: somatostatin receptor; TAE: trans-arterial embolization; TE: telotristat etiprate; TEM: temozolomide; WD-SINENs: well-differentiated small intestinal neuroendocrine neoplasms.

Importantly, the mainstay of treatment for MF in SI-NEN patients is locoregional resective surgery either with a curative intent or to provide symptomatic relief, depending on the stage of the disease (Figure 4) [87]. However, compromise of the mesenteric vasculature in the root of the mesentery may render surgical resection technically challenging and endanger the circulation to substantial parts of the intestine, leading to devastating complications such as short-bowel syndrome. Nevertheless, radical resection is not always possible due to the location of large metastatic LN conglomerates and the encasement of the superior mesenteric vessels in the root of the mesentery [5,79]. The National Comprehensive Cancer Network Guidelines on SI-NENs advocate against the resection of a small, asymptomatic, relatively stable primary tumor in the presence of unresectable metastatic disease. A recent study has confirmed no OS benefit in asymptomatic patients with distant-stage disease who underwent upfront locoregional resective surgery, however radiological assessment of MF was not included [88]. These results are also consistent with the findings of two recent cohort studies of SI-NEN patients with MF that confirmed no survival benefit of locoregional resective surgery in the presence of MF in asymptomatic patients with stage IV disease [85,89]. However, a further study has revealed that the presence of MF is linked with a more aggressive biological behavior when PFS was used as a surrogate endpoint, despite similar Ki-67 labelling indices [90]. In addition, size and morphologic features of mesenteric tumor deposits appear to have no prognostic impact; instead, deposit multifocality may be associated with patient outcomes, whereas the extent of bowel resection seems to be associated with the extent of MF [91,92]. A significant association of MF with metastatic patterns and with criteria of functionality has been confirmed, suggesting a common pathophysiological mechanism [86]. It should be noticed though that while asymptomatic patients with MF and disseminated, SI-NENs may not benefit from up-front prophylactic surgery; some patient populations might, such as older patients and patients with progressive loco-regional disease [93]. Importantly, in order to be able to identify patients who might benefit from a prophylactic surgical approach, more insight is needed into the development of MF in SI-NENs [94]. Of note, multivisceral transplantation including the small intestine is a seldom-utilized approach worldwide for highly selected patients with extensive metastatic burden, i.e., potentially some SI-NEN patients with no liver metastases but extensive metastatic disease in the root of the mesentery and associated MF threatening a vascular catastrophe of the gut by encasement of mesenteric vessels [95].

##### Minimal Invasive Management

SI-NEN patients with an advanced degree of arterial and venous involvement in the root of the mesentery and in the presence of extensive MF are unlikely to receive radical locoregional surgery and should therefore be spared unnecessary laparotomies if asymptomatic [91]. Interestingly, prolonged OS has been demonstrated after minimal invasive interventions in patients with inoperable locoregional disease due to MF, as compared to those with no intervention and a similar stage IV tumor burden [5]. Transhepatic endoluminal stenting of the superior mesenteric vein appears to be a relatively safe procedure that may be undertaken in cases with advanced inoperable disease and severe ischemic symptoms resistant to conservative medical control [5,97]. Stent failure is most likely attributed to thrombosis and the role of anticoagulation in this setting remains to be defined in further studies with longer follow-up [96]. Other minimal invasive measures with palliative purposes in order to preserve renal function in advanced stages may include intra-ureteral J-stent and percutaneous nephrostomy for the management of obstructive uropathy due to advanced SI-NEN-related retroperitoneal fibrosis [5].

#### 3.4.2. Medical Treatment

##### Somatostatin Analogues (SSAs)

The CLARINET and PROMID trials have shown that long-term treatment with long-acting somatostatin analogues (SSAs), lanreotide autogel and octreotide slow release, may exert an anti-tumor activity on SI-NENs [98,99]. Treatment with SSAs designed to reduce circulating tumor metabolites (including 5-HT), has been shown to achieve symptomatic and antitumoral response and may confer the additional advantage of retarding or preventing the progression of CHD [100]. In addition, assessment of the level of serum fibrosis markers CTGF and TGFβ in patients with CS treated with prolonged-release SSAs revealed a reduction of CTGF and TGFβ levels, which could in turn prevent the formation of MF [30]. This study confirmed other preclinical ones exhibiting a potential inhibitory effect of SSAs in local fibrosis formation by reducing the secretion of profibrotic growth factors such as TGFβ [101,102]. Despite the control of CS, including normalization of serotonin secretion and reduction of bioactive peptides, the effect of SSAs in MF remains to be determined prospectively in further studies.

##### Serotonin Synthesis Inhibitors and 5-HT Receptor Antagonists

As the FGF pathway is highly activated in SI-NENs with CS owing to elevated serotonin, it has been implied that therapies that inhibit serotonin synthesis may prevent CHD and MF [71,103]. Efficacy of telotristat ethyl, a novel agent inhibiting peripheral serotonin synthesis was assessed in two phase III clinical trials: TELESTAR and TELECAST [104,105]. This prodrug is converted into its active metabolite: telotristat etiprate that inhibits tryptophan function, resulting in a reduction of the serotonin bloodstream levels [106]. The TELESTAR trial reported a reduction in diarrhea in those patients treated with telotristat ethyl together with a significant reduction of urinary 5-HIAA levels [104]. The TELECAST study provided further confirmation about the efficacy and the safety of telotristat ethyl [105]. With respect to the impact of telotristat ethyl on fibrosis, two of the patients recruited into the TELESTAR trial with known CHD had stable valvular function on follow-up [106,107]. However, no evidence of antitumor effect or impact of telotristat ethyl on MF is available as yet.

Apart from inhibition of 5-HTsynthesis, 5-HT receptor antagonists may target serotonin signaling and potentially reduce MF in SI-NENs. The 5HT2 antagonists ketanserin and cyproheptadine have been found to inhibit attacks of flushing, diarrhea and dyspnea in patients with CS [108,109]. Their therapeutic benefit would appear to be a peripheral effect, as 5-HIAA excretion in these patients was not reduced [109]. Another 5-HT receptor antagonist, terguride, reduces the pro-fibrotic potential in scleroderma and suppresses pathways implicated in the regulation of pro-fibrotic genes, suggesting that 5-HT inhibitors might reduce MF via suppression of TGF-β1-mediated non-canonical signaling pathways [110]. Therefore, serotonin synthesis antagonists and 5-HT receptor inhibitors may have an important role not only in the management of severe CS, but also in the prevention of MF, pending confirmation of prospective clinical studies in SI-NENs.

##### Molecular Targeted Therapies (mTOR and Tyrosine Kinase Inhibitors)

The phase III RADIANT-4 trial reported that everolimus significantly improved PFS in patients with progressive SI-NENs [111]. However, the response to treatment is not durable, most likely due to incomplete and unsustained inhibition of mTORC1 signaling by this compound, and/or activation of mTOR complex 2 (mTORC2), underscoring the need for alternative therapies and drug combinations. In a recent preclinical study, in vitro mTOR kinase inhibition effectively stabilized progressive NENs and delayed cardiac impairment [112]. mTOR inhibition may result in a decrease of the expression of 5-HT receptors, as well as that of other players that may be implicated in fibrosis development: however, the exact mechanisms underlying mTOR inhibition’s impact on MF are currently unknown [112,113].

SI-NEN proliferation is responsive to a number of GFs, such as EGF, PDGF, TGFα, TGFβ and CTGF, and therefore may be susceptible to tyrosine kinase inhibitors (TKIs) targeting these receptors or associated signaling pathways [23,114,115]. In particular, TGFα stimulation can be inhibited at several points of the MAPK pathway, but success is limited to NEN models and is not evident in the clinical setting. In addition, although some NENs are inhibited by TGFβ1, paradoxical growth has been seen in experimental models of SI-NENs [114]. CTGF expression is associated with more malignant clinical phenotypes, as it promotes growth in SI-NEN models, and is implicated as a mediator of local and distant fibrosis. However, the anti-proliferative effect of CTGF inhibition has not been tested in NENs [114]. Notably, although TKIs have not gained approval and their efficacy seems limited in SI-NENs; their impact on MF and CHD have not been specifically addressed [116,117]. Contemporary research on fibrotic diseases focuses on promising TKIs, such as imatinib-targeting c-alb kinases and PDGF receptors [118,119]. The multiple and overlapping signaling pathways that characterize SI-NENs suggest targeting these tumors at a number of levels may be required to provide efficacy [114]. Although the studies assessing the efficacy of TKIs in SI-NENs did not focus on MF, the molecular pathways involved in its development are similar between SI-NENs and other fibrotic conditions that TKIs can target. Thus, the use of TKIs in SI-NENs could be extended beyond their anti-tumor effects and also be evaluated in the context of prevention or reversal of MF.

##### Peptide Receptor Radionuclide Therapy (PRRT)

The efficacy of PRRT in the management of SI-NENs has been confirmed in the NETTER-1 trial [120]. The delivery of targeted PRRT locoregionally in the mesenteric LN conglomerate could be associated with a risk of inducing a local reaction, hence increasing the risk of MF-related complications. In a recent study investigating this, the severity of MF did not seem to be affected after PRRT [121].

##### Other Antifibrotic Agents

Angiotensin-converting-enzyme (ACE) inhibitors through renin–angiotensin system inhibition may reduce tumor progression and potentially affect SI-NEN-related MF [122]. Ex vivo angiotensin-converting enzyme (ACE) inhibition has been shown to reduce BON1 cell proliferation, but further in vivo and human studies are needed to address ACE inhibitors’ clinical efficacy in patients with SI-NENs [28]. In addition, tamoxifen has been used in SI-NENs in the 1980s for control of CS and also for that of tumoral growth [33,34,35,123]. More recently, it has been used in other fibrotic diseases, however its clinical value in MF, probably mediated by TGFβ inhibition, has not been determined as yet [124,125].

## 4. Conclusions

SI-NENs are often associated with the development of locoregional fibrotic reactions in the mesentery that may lead to severe symptomatology with significant associated morbidity. Locoregional resective surgery remains the mainstay of treatment in symptomatic cases of all stages, however its survival benefit in asymptomatic patients with disseminated disease has been recently challenged. Moreover, in inoperable cases, prompt recognition of MF-related complications and minimally invasive interventions as clinically indicated per patient seem effective in disease palliation. As the impact of modern multimodality systemic and/or targeted treatments in SI-NEN-related MF has not been adequately investigated, elucidation of the molecular basis of MF in this setting may guide our ability to develop predictive biomarkers as well as targeted drug therapies for the prevention or reversal of MF. Three-dimensional organoids/spheroids and coculture of these with other cell types may be a promising model for several translational applications that may facilitate the study of NEN stroma complexity and its influence in epithelial tumorigenesis in the context of MF. In addition, the investigation of epigenetic changes in SI-NEN as a driver of MF and the clinical utility of profibrotic circulating transcripts as a novel biomarker for MF stratification should be assessed in further studies. Finally, composite endpoints in SI-NEN future clinical studies, such as health-related quality of life, symptom-specific survival or development of symptoms specifically owing to growing mesenteric LN metastases and/or increasing MF may be more valid than OS and PFS as endpoints when assessing the effects of SINEN-related MF. Although the antitumor efficacy of TKIs and 5-HT synthesis inhibitors and receptor antagonists in SI-NENs may be limited or may not have been fully determined respectively, further studies on these drugs and other novel agents targeting MF are warranted to guide the development of medical treatments that would reduce MF and alleviate associated symptoms.

## Figures and Tables

**Table 1:** Most studied profibrotic mediators secreted by small intestinal neuroendocrine neoplasms (SI-NENs) and their implications as potential therapeutic targets related to mesenteric fibrosis (MF).

Fibrotic Factors	Specific Invovement in MF	Potential Therapeutic Agent Targenting MF	Relevant Studies
Serotonin	-mitogenic effect in stroma cells -cyclin E induction -potentiates the effects of PDGF, β-FGF, EGF and insulin -decreased expression of serotonin degrading enzymes in the stroma	-SSAs -5-HT synthesis inhibitors and receptor antagonists -Tyrosine kinase inhibitors	[12] [14] [22] [23] [24] [25] [26] [27]
TGF-β	-chemotactic for fibroblasts and macrophages -cell proliferation -production of extracellular matrix and stimulation of growth factor secretion by fibroblasts	-SSAs -Tyrosine kinase inhibitors -ACE inhibitors -Tamoxifen	[28] [29] [30] [31] [32] [33,34,35] [36]
CTGF	-promoter of mitosis, chemotaxis -stimulator of apoptosis, angiogenesis, synthesis of collagens, fibronectin and α5-integrin -transcriptional activation through TGF-β1 and other mediators acting in turn as downstream mediator on fibroblasts -SMAD, PKC and ras/MEK/ERK (kinase) pathways necessary for the TGFβ1-mediated induction of the CTGF promoter	-SSAs -Tyrosine kinase inhibitors	[37] [38] [30] [10,39,40,41]
PDGF	-mitogenic properties on fibroblasts -PDGF a-receptor seen on clusters of tumour cells and occasionally on adjacent stroma -PDGF β -receptor seen only in the stroma -cyclin D1 expression via the MAPK/ERK pathway	-Tyrosine kinase inhibitor (imatinib)	[42] [43] [13] [44]
IGF-1	-cell proliferation -mitogenic effect on fibroblasts -serotonin-IGF-1 axis activation	-5-HT synthesis inhibitors and receptor antagonists	[45] [46] [36] [37] [47] [48]
EGF	-cell proliferation and differentiation -highly expressed in SI-NENs	-Tyrosine kinase inhibitors	[46]
TGF-α	-interaction with EGF EGFR receptor activation -highly expressed in SI-NENs	-Tyrosine kinase inhibitor	[46] [49] [47]
FGF	-potent stimulant of endothelial cell growth -cell proliferation and stroma formation	-Tyrosine kinase inhibitors	[12] [28] [36] [43]
VEGF	-secreted by CAFs to induce tumour cell proliferation	-Tyrosine kinase inhibitors	[46] [50] [6]
NGF	-regulatory effects on angiogenesis -highly expressed in mesenteric angiopathy	-Tyrosine kinase inhibitors	[36]
VAP-1	-higly expressed in SI-NEN stroma	-Pyridazone inhibitors -hVAP-1-targeted inhibitors	[51] [52]
BMP	-members of the TGF-β superfamily -cell growth and differentiation -promoter of angiopathy	-small molecule inhibitor of BMP signaling, K02288 -Statins	[51] [53] [54,55]

Abbreviations: 5-HT: serotonin; ACE: angiotensin-converting enzyme; BMP: bone morphogenic protein; CAFs: cancer-associated fibroblasts; CTGF, connective tissue growth factor; EGF: epidermal growth factor; EGFR: epidermal growth factor receptor; ERK: extracellular-signal-regulated kinase; FGF2, fibroblast growth factor 2; β-FGF: fibroblast growth factor beta; hVAP-1: human vascular adhesion protein-1; IGF-1: Insulin-like growth factor 1; K02288: bone morphogenetic protein (BMP) type I receptor inhibitor; MAPK: Mitogen-activated protein kinase; MEK: Mitogen-activated protein; MF: mesenteric fibrosis; PDGF: platelet-derived growth factor; PKC: Protein kinase C; SI-NEN, small intestinal neuroendocrine neoplasm; SMAD: an acronym from the fusion of Caenorhabditis elegans Sma genes and the Drosophila Mad, Mothers against decapentaplegic; SSAs: somatostatin analogues; TGFα: transforming growth factor alpha; TGFβ, transforming growth factor beta.

## Supplementary Materials

The following are available online at https://www.mdpi.com/2077-0383/9/6/1777/s1, Table S1: Systematic Literature Search Strategy.
