# Supplementary material for: Pathogenesis and Clinical Management of Mesenteric Fibrosis in Small Intestinal Neuroendocine Neoplasms: A Systematic Review
Source: J Clin Med. 2020 Jun 8;9(6):1777. doi: 10.3390/jcm9061777 (PMC7357094; doi:10.3390/jcm9061777)
Supplement: Supplementary file 1 [file jcm-09-01777-s001.pdf]

Embase 200122

	Search terms.	Number of studies
	**Neuroendocrine tumors**	
**1.**	(neuroendocrine NEAR/3 tumo*r*):ti,ab,kw OR (neuroendocrine NEAR/3 cancer*):ti,ab,kw OR (neuroendocrine NEAR/3 neoplas*):ti,ab,kw OR (neuroendocrine NEAR/3 carcinom*):ti,ab,kw OR (neuroendocrine NEAR/3 malignanc*):ti,ab,kw OR (adenoma* NEAR/1 chromophobe):ti,ab,kw OR (adenoma* NEAR/1 basophil):ti,ab,kw OR (adenoma* NEAR/1 acidophil):ti,ab,kw	**36,732**
**2.**	'neuroendocrine tumor'/de OR 'gastroenteropancreatic neuroendocrine tumor'/exp OR 'multiple endocrine neoplasia type 1'/de OR 'chromophobe adenoma'/de OR 'neuroendocrine carcinoma'/exp OR 'vipoma'/de OR 'somatostatinoma'/de OR 'multiple endocrine neoplasia type 1':ti,ab,kw OR 'men 1':ti,ab,kw OR vipoma*:ti,ab,kw OR somatostatinoma*:ti,ab,kw OR apudoma*:ti,ab,kw	**54,095**
**3.**	'carcinoid'/de OR 'carcinoid syndrome'/de OR 'gastrointestinal carcinoid'/exp OR carcinoid*:ti,ab,kw	**26,212**
**4.**	1. OR 2. OR 3.	**82,367**
	**Fibrosis**	
**5.**	'fibrosis'/de OR 'intestinal fibrosis'/de OR 'peritoneal fibrosis'/de OR 'retroperitoneal fibrosis'/de OR 'tissue adhesion'/de OR 'sclerosis'/de OR 'desmoplasia'/de OR 'fibroplasia'/de OR fibrosis:ti,ab,kw OR fibroses:ti,ab,kw OR fibrotic:ti,ab,kw OR fibroplasia:ti,ab,kw OR sclerosis:ti,ab,kw OR scleroses:ti,ab,kw OR sclerotic:ti,ab,kw OR desmoplasia:ti,ab,kw OR 'desmoplastic reaction*':ti,ab,kw OR (tissue NEAR/1 adhesion*):ti,ab,kw	**530,263**
	**Combined sets**	
**6.**	4. AND 5.	**1,272**
	**After deduplication**	**779**

Web of Science 200122

	Search terms.	Number of studies
	**Neuroendocrine tumors**	
**1.**	**TOPIC:** (neuroendocrine NEAR/3 tumo*r*) OR **TOPIC:** (neuroendocrine NEAR/3 cancer*) OR **TOPIC:** (neuroendocrine NEAR/3 neoplas*) OR **TOPIC:** (neuroendocrine NEAR/3 carcinom*) OR **TOPIC:** (neuroendocrine NEAR/3 malignanc*) OR **TOPIC:** (adenoma* NEAR/1 acidophil) OR **TOPIC:** (adenoma* NEAR/1 basophil) OR **TOPIC:** (adenoma* NEAR/1 chromophobe) OR **TOPIC=(“**multiple endocrine neoplasia type 1” OR "MEN 1" OR vipoma* OR somatostatinoma* OR apudoma* OR carcinoid*)	**42,882**
	**Fibrosis**	
**2.**	**TOPIC=**(fibrosis OR fibroses OR fibrotic OR fibroplasia OR sclerosis OR scleroses OR sclerotic OR desmoplasia OR “desmoplastic reaction*”) OR **TOPIC:** (tissue NEAR/1 adhesion*)	**454,040**
	**Combined sets**	
**3.**	1. AND 2.	**622**
	**After deduplication**	**209**

Medline through Ovid 200124

	Search terms.	Number of studies
	**Neuroendocrine tumors**	
**1.**	(neuroendocrine adj4 (tumo?r* or neoplas* or cancer* or carcinom* or malignanc*)).ab,kf,ti.	**21,066**
**2.**	neuroendocrine tumors/ or adenoma, acidophil/ or adenoma, basophil/ or adenoma, chromophobe/ or apudoma/ or carcinoid tumor/ or malignant carcinoid syndrome/ or carcinoma, neuroendocrine/ or somatostatinoma/ or vipoma/ or Multiple Endocrine Neoplasia Type 1/	**27,571**
**3.**	(carcinoid* or somatostatinoma* or vipoma* or apudoma* or (adenoma* adj2 chromophobe) or (adenoma* adj2 basophil*) or (adenoma* adj2 acidophil*) or "Multiple Endocrine Neoplasia Type 1" or "MEN 1").ab,kf,ti.	**20,054**
**4.**	1. OR 2. OR 3.	**43,080**
	**Fibrosis**	
**5.**	(fibrosis or fibroses or fibroplasia or desmoplasia or sclerosis or scleroses or fibrotic or sclerotic or "desmoplastic reaction" or "desmoplastic reactions" or (tissue adj2 adhesion*)).ab,kf,ti.	**333,020**
**6.**	fibrosis/ or tissue adhesions/ or peritoneal fibrosis/ or retroperitoneal fibrosis/	**44,050**
**7.**	exp Sclerosis/	**8,712**
**8.**	5. OR 6. OR 7.	**355,024**
	**Combined sets**	
**9.**	4. AND 8.	**554**
	**After deduplication**	**549**

Scopus 200123

	Search terms.	Number of studies
	**Neuroendocrine tumors**	
**1.**	(TITLE-ABS-KEY ((neuroendocrine W/3 (tumo*r* OR neoplas* OR cancer* OR carcinom* OR malignanc*)))) OR (TITLE-ABS-KEY (((carcinoid* OR somatostatinoma* OR vipoma* OR apudoma* OR (adenoma* W/1 chromophobe) OR (adenoma* W/1 basophil*) OR (adenoma* W/1 acidophil*) OR "Multiple Endocrine Neoplasia Type 1" OR "MEN 1"))))	**54,719**
	**Fibrosis**	
**2.**	TITLE-ABS-KEY ((fibrosis OR fibroses OR fibroplasia OR desmoplasia OR sclerosis OR scleroses OR fibrotic OR sclerotic OR "desmoplastic reaction" OR "desmoplastic reactions" OR (tissue W/1 adhesion*)))	**505,999**
	**Combined sets**	
**3.**	1. AND 2.	**1,097**
	**After deduplication**	**355**

Cochrane 200124

	Search terms.	Number of studies
	**Neuroendocrine tumors**	
1.	((neuroendocrine NEAR/3 (tumo?r* or neoplas* or cancer* or carcinom* or malignanc*))):ti,ab,kw	1036
2.	((carcinoid* or somatostatinoma* or vipoma* or apudoma* or (adenoma* NEAR/1 chromophobe) or (adenoma* NEAR/1 basophil*) or (adenoma* NEAR/1 acidophil*) or "Multiple Endocrine Neoplasia Type 1" or "MEN 1")):ti,ab,kw	694

3.	MeSH descriptor: [Neuroendocrine Tumors] this term only	145
4.	MeSH descriptor: [Adenoma, Acidophil] this term only	0
5.	MeSH descriptor: [Adenoma, Basophil] explode all trees	0
6.	MeSH descriptor: [Adenoma, Chromophobe] this term only	1
7.	MeSH descriptor: [Apudoma] this term only	0
8.	MeSH descriptor: [Carcinoid Tumor] this term only	72
9.	MeSH descriptor: [Malignant Carcinoid Syndrome] this term only	34
10.	MeSH descriptor: [Carcinoma, Neuroendocrine] this term only	38
11.	MeSH descriptor: [Somatostatinoma] this term only	3
12.	MeSH descriptor: [Vipoma] this term only	2
13.	MeSH descriptor: [Multiple Endocrine Neoplasia Type 1] this term only	9
14.	1. OR 2. OR 3. OR 4. OR 5. OR 6. OR 7. OR 8. OR 9. OR 10. OR 11. OR 12. OR 13.	1521
	**Fibrosis**	
15.	((fibrosis or fibroses or fibroplasia or desmoplasia or sclerosis or scleroses or fibrotic or sclerotic or "desmoplastic reaction" or "desmoplastic reactions" or (tissue NEAR/1 adhesion*))):ti,ab,kw	27345
16.	MeSH descriptor: [Fibrosis] this term only	1254
17.	MeSH descriptor: [Tissue Adhesions] this term only	456
18.	MeSH descriptor: [Peritoneal Fibrosis] this term only	3
19.	MeSH descriptor: [Retroperitoneal Fibrosis] this term only	6
20.	MeSH descriptor: [Sclerosis] this term only	969
21.	15. OR 16. OR 17. OR 18. OR 19. OR 20.	27345
	**Combined sets**	
22.	14. AND 21.	17
	**After deduplication**	4
